# Supporting Reassigned Hospital Staff During the COVID-19 Pandemic in the Montreal Region: What Does it say About Leadership Styles?

**DOI:** 10.1177/08445621231192044

**Published:** 2023-08-17

**Authors:** Lara Gautier, Morgane Gabet, Arnaud Duhoux, Lola Traverson, Valéry Ridde, Kate Zinszer, Pierre-Marie David

**Affiliations:** 1 School of Public Health, University of Montréal, Montréal, Canada; 2 Centre de recherche en santé publique (CReSP), 248214 University of Montréal and CIUSSS du Centre-Sud-de-l’Île-de-Montréal, Montréal, Canada; 3 Université Paris Cité, 27056 IRD, Inserm, Ceped, F-75006 Paris, France; 4 63678 Faculty of nursing, University of Montréal, Montréal, Canada; 5 63677 Faculty of pharmacy, University of Montréal, Montréal, Canada

**Keywords:** health human resources, leadership, occupational health, Quebec

## Abstract

Globally, the COVID-19 pandemic took a high toll on health human resources, especially in contexts where these resources were already fragile. In Quebec, to make up for the shortage of health human resources, and to contain the COVID-19 outbreaks in long-term care facilities, many hospital staff (including a majority of nurses) were sent to those facilities, with varying degrees of support. Building on the body of evidence linking leadership style and resilience, we conducted a qualitative comparative analysis of two hospitals in the Montreal Metropolitan Area, Quebec. We explored respondents’ experience of psychosocial support tools provided to hospital staff reassigned to COVID-affected facilities. Data from 27 in-depth interviews with high- and mid-level managers, and front-line workers, was analyzed through the lens of leadership styles. Our findings highlighted how the design and implementation of support tools revealed major differences across the two hospitals’ leadership styles (i.e., one hospital expressing leader-centered styles vs. the other expressing follower-centered leadership styles). The expression of these leadership styles was largely shaped by recent policies, notably a major political reform of 2015, which enforced more centralized decision-making. Our study offered additional empirical evidence that leadership styles fostering the recovery of health human resources may be a key indicator of successful response to crises.

## Introduction

Health care facilities are at the forefront of crisis response, being the first point of contact for triage, detection, and treatment of populations (Denis et al., 2021). In hospitals and other health care organizations, the daily routines of health human resources (HHR) were thus significantly impacted by COVID-19 – which in turn affected their capacity to cope with a crisis. In addition, to contain COVID-19 outbreaks, several governments issued orders to allow hospitals to send their staff to most-affected services and facilities, an organizational phenomenon hereinafter referred to as “reassignment”. This decision entailed profound changes for the reassigned staff, with significant consequences for their wellbeing at work (Khajuria et al., 2021).

Previous studies have shown that, during a crisis, leaders play a central, crucial role to support staff's wellbeing at work and resilience (Grint, 2020; Obrien et al., 2021). Leadership has been defined as “the relationship between the individual/s who lead and those who take the choice to follow, while it refers to the behavior of directing and coordinating the activities of a team or group of people towards a common goal” (Sfantou et al., 2017, p. 1). Based on this definition, several leadership styles co-exist, and among them, some have specific features which may help organizations navigate through crises. A limited number of studies have investigated the relationships between leadership styles of health care organizations and/or managers, and the organizations’ or managers’ preparedness or response adequacy towards HHR. While several studies quantitatively and qualitatively described the reassignment experience (Juan et al., 2022; Kennedy et al., 2022), there is to our knowledge no literature reflecting on the phenomenon of reassignment in relation to leadership styles in the context of the response to the COVID-19 crisis.

Leadership styles can be classified into two categories, namely leader-centered styles, and follower-centered styles. According to Maslennikova, “leader-centered styles achieve organizational success through the self-realization and self-projection of the leader” (Maslennikova, 2007, p. 1). Common leader-centered styles include *transactional leadership*, *authoritative leadership*, and *laissez-faire leadership.* These styles are more often found in organizations that have top-down, hierarchical governance structures. Leaders considered ‘laissez-faire’ are often depicted as lacking involvement during critical organizational junctures (Eagly et al., 2003). Authoritarian leaders are task-oriented figures that provide their followers with clear expectations of what needs to be accomplished, and how it should be accomplished (Lewin et al., 1939). Transactional leaders promote followers’ compliance to a rewards and punishments system, which keeps followers motivated for the short-term (Bass, 1985). This type of leadership is said to be effective in emergency situations because they “share a concern for the resolution of immediate needs” (Tomkins & Simpson, 2015, p. 1020). Follower-centered styles reflect leadership styles that recognize and promote employees as valuable organizational assets, deserving careful attention (Maslennikova, 2007). Organizations that allow for follower-centered styles to express have horizontal governance structures. The most common follower-centered style is *transformational leadership*. Transformational leaders empower, inspire, and motivate followers to innovate and create change in organizations through a collective vision and greater ownership of their work (Bass, 1985). Transformational leaders generally are empathetic leaders. Unlike *transactional leadership*, transformational leadership reached beyond the immediate needs (e.g., in times of crisis) and often involves lengthy temporal trajectories to achieve long-term inspirational goals (Tomkins & Simpson, 2015). Other follower-centered styles include *inclusive leadership* and *servant leadership*. Inclusive leadership refers to a set of leader efforts to invite and appreciate group members’ contribution, while enhancing their feeling of being part of the group (belongingness) (Nembhard & Edmondson, 2006). This leadership style has been explored in the context of the pandemic, thereby showing the influence of inclusive leadership style in reducing the psychological distress of nurses during the COVID-19 crisis in Wuhan (Zhao et al., 2020). The goal of *servant leaders* is to serve their followers; they interact with them to achieve authority rather than power (Allen et al., 2016). Servant leaders focus on staff wellbeing. Lastly, by suggesting to rise above the leader-follower dichotomy, Tomkins & Simpson's definition of *caring leadership* goes beyond merely kind or compassionate leaders by re-emphasizing human agency and the consciousness of staff (Tomkins & Simpson, 2015). Caring leaders can navigate complex organizational decision-making with flexibility and adaptability – which we might also consider assets of organizational resilience.

This study is grounded in transformational leadership theory. According to this theory, leadership's effects and impacts are typically revealed and represented through the eyes of an organization's staff (Bass, 1985). Staff's perceptions of leadership's actions thus provide evidence of leadership effectiveness. Assessing the latter requires a detailed understanding of how leaders’ decisions were made and how those decisions’ implementation and effects were perceived by staff. Several studies described the effects of transformational leadership sub-categories, such as compassionate managerial leadership (Oruh et al., 2021) and/or adaptative leadership (Laur et al., 2021) on health care organizations’ crisis response. Among these studies, none conceptualized leadership styles in the context of specific health care organizations. Wibowo and Paramita found positive associations between empathetic leadership and COVID nurses’ resilience in Indonesia (Wibowo & Paramita, 2021). However, this work was not carried out at the organizational level and hence did not account for specific organizational decisions. To fill the research gap, our study investigated how two contrasted hospitals in Quebec supported HHR during the first and second waves of the pandemic (i.e., March-June 2020, and September 2020 to February 2021). Quebec was the Canadian province that experienced the highest COVID-19 death rate during the first two waves of the pandemic (Canada, 2020). Quebec's experience is significant because, in addition to an inadequate geographic distribution of HHR (Ministère de la Santé et des Services sociaux, 2022), the second most-populated Canadian province has had recurring challenges in retaining HHR. Reasons for lack of retention include the impacts of previous health system reforms, and a recurring lack of flexibility for accommodating family/work balance (Dubois, 2020). Bill 10 is a key element to understanding hospital (re)organization in Quebec. It primarily relied on administrative institutional integration and centralization of power at the macro-political (provincial) level. Table 1 below outlines the contents of Bill 10 and its influence on how reassignment decisions were made and how it impacted organizations and HHR.

**Table 1.** Bill 10, the reorganization of health and social services, and their influence on reassignment decision-making during the COVID-19 crisis.

Socio-political levels	Contents of Bill 10 and its implications for health and social services reorganization	Direct and indirect influence of Bill 10 on reassignment decision-making, implementation, and impacts, during COVID-19
Macro-level (provincial level)	Bill 10 induced more centralized decision-making, by abolishing regional health authorities, by instituting a direct report of CI(U)SSS to the minister, by replacing democratic processes for electing institutional boards of directors with government-appointed boards. For example, instead of being appointed by executive boards and through democratic processes, chief executive officers (CEOs) of CI(U)SSS became in 2015 directly appointed by the ministry of health, thus granting the latter significant leverage over the organization of CI(U)SSS.	March-April 2020 – issuance of ministerial orders and communiqués instituting staff reassignment: Order number 2020-007 of the MSSS dated March 21, 2020, which sees the modification of collective agreements and the possibility of reassignment of employees in the health network; Order number 2020-019 dated April 10, 2020, which gives the possibility of reassigning personnel from the education network to the health and social services network.
Meso-level (organizational level)	Bill 10 created 22 integrated health and social services networks (CISSS) (e.g., our case C2), nine of which designated “university integrated health and social services networks” (CIUSSS) and seven non-integrated hospitals (e.g., our case C1). The CI(U)SSS are responsible for service delivery and some degree of planning, thereby responding to population health needs in their respective territory. This reorganization translated into the *de facto*, streamlining of several health and social facilities, merged into large CI(U)SSS. Renewed focus on hospitals and clinical care, at the expense of long-term care services.	In all CI(U)SSS and non-integrated hospitals, training sessions were offered to staff, to increase their competence in intensive care. Some hospitals also offered basic training in geriatrics, with the goal of sending these newly trained hospital staff to long-term care settings. In the whole health and social services network, reassignment policies were initially *voluntary*; financial incentives were offered to those reassigned. In CI(U)SSS located in the most COVID-affected areas of Quebec, the application of ministerial orders quickly prompted *mandatory* reassignment, which was not the case for non-integrated (autonomous) hospitals (e.g., C1).
Micro-level (individual/team- level)	More than 20% middle managers were cut as a result of Bill 10, which meant less communications with clinical teams under their responsibility in the various organizations part of CI(U)SSS. This issue widened the distance between clinical teams and executive teams – which in turn impacted psychological distress at work, and resulted in increased absenteeism for clinical staff. Staff shortages (for both clinical and administrative staff) in CI(U)SSS thus predated the pandemic, including in long-term care settings under the purview of CI(U)SSS.	In March-April 2020, many employees working in long-term care settings became infected with COVID-19; their absence led to a massive abandonment of residents, leaving facilities even more vulnerable to COVID-19 clusters. CI(U)SSS staff – who were already vulnerable because of the impacts of Bill 10, and particularly those who were reassigned, were likely to experience: workplace anxiety – especially those who were reassigned to long-term care facilities; fear of being infected with COVID-19; professional exhaustion; loss of professional meaning.

In the context of this reform, at the organizational level (meso-level), middle-management support structures for front-line workers within health care facilities were also streamlined. More than 20% middle managers were cut as a result of this reform, which meant less communication with clinical teams under their responsibility (Denis et al., 2021). According to various analysts, the bill's vision change was done at the expense of public health, long-term care services, and social services (Quesnel-Vallée & Carter, 2018). Indeed, Bill 10's application also translated into a renewed focus on hospitals, which are the bigger and most expensive components in the newly-created integrated networks (CISSS and CIUSSS). Within hospitals, a stronger centralization of power, and the cutting of middle managers, increased the distance between clinical teams and executive teams. At the individual level (micro-level), one plausible consequence of this issue is increasing psychological distress at work. In 2019, nearly 40% of HHR absenteeism in the health care sector was caused by mental health issues (Ministère de la Santé et des Services sociaux, 2019). A fragile HHR situation thus predated the health crisis; it contributed to worsening HHR shortages. Many HHR in long-term care settings became infected with COVID-19, and their absenteeism led to a massive abandonment of residents, leaving facilities even more vulnerable to COVID-19 clusters (Commissaire à la santé et au bien-être Québec, 2020). In fact, 62.6% of all COVID-19 deaths in Quebec in 2020 were identified in long-term care facilities (Institut National de Santé Publique du Québec, 2020). To make up for such HHR shortage and to contain COVID-19 outbreaks in long-term care facilities, many hospital staff were sent to those facilities, with significant consequences for their mental health (Cyr et al., 2021). Building upon their observational study findings, Cyr and colleagues invite scholars to investigate perceptions of organizational support tools. Drawing on the unique experience of HHR reassignment in Quebec, we decided to investigate the staff's perceptions of organizational support tools provided by high-level and mid-level managers from the ‘home’ hospitals.

Based on a comparative analysis of two hospitals in the Montreal Metropolitan Area, Quebec, this study sought to answer the following research question: *What leadership styles are reflected through the hospitals’ support responses for hospital staff reassigned to long-term care services during the first and second pandemic waves in the Montreal region?* To answer this question, we used a qualitative case study documenting hospital staff's experience with reassignment decisions and their perceptions of tools implemented to support staff that went to COVID-affected long-term care facilities (LTCFs).

## Methods

### Study design and case selection

This research was embedded in a larger multicountry research project entitled *HoSPiCOVID*, on hospitals’ and health systems’ resilience in the context of COVID-19 (Ridde et al., 2021). The present study relies on two contrasted case studies (Yin, 2009), two hospitals (C1 and C2) within the Montreal Metropolitan Area, in Quebec (Canada).

We selected two pandemic-hit health care organizations in Quebec that would reflect a differentiated experience of the COVID-19 crisis, primarily based on geographical and institutional contrasting criteria. These selected hospital cases in Quebec, located in two of the most affected areas in the province, indeed represented contrasting organization types as a result of Bill 10, regarding their respective institutional integration, the health territories covered, and their respective scope of intervention (Case 1 being a single, autonomous, specialized, pediatric hospital; Case 2 being a general hospital, integrated in a regional network, and overseeing many different health care facilities falling under its catchment area), as explained below. For the specific purpose of the present paper, our case selection enabled us to highlight how several leadership styles could be revealed through a) key organizational governance differences (i.e., C1 has an autonomous organizational structure vs. C2 is governed by a CISSS reporting directly to the ministry), and b) diverse approaches to reassignment (e.g., where reassignment was offered on a voluntary basis in C1; vs. when it was mandatory in C2) and perceptions of support tools offered to reassigned staff.

### Study setting

In Quebec, at the beginning of the first wave, the priority was to preserve hospitals against anticipated mass arrivals of COVID-19 patients. All efforts were focused on protecting hospitals and ensuring that they had sufficient staff. On March 10^th^, 2020, public authorities realized how much the pandemic was affecting the long-term care sector. A government communiqué recommended that hospitals send staff to long-term care facilities which are overseen by their respective CISSS/CIUSSS, thus shifting the focus back to these long-term care settings that were facing dramatic outbreaks of COVID-19. Both hospitals in the present study were directly impacted by this major change in situation.

### Cases description

The first case (C1) is a Montreal hospital specializing in pediatrics. It is the largest pediatric facility in Canada and one of the four largest pediatric centers in North America and has 550 beds. As of 2021, C1 had 6,383 employees, including 1,757 nurses and nursing assistants, as well as 574 physicians, dentists, and pharmacists (Sainte-Justine, & Lavoi, n.d.). Unlike the large majority of hospitals in Quebec, which were the subject of the vast merging operation of Bill 10 (Larivière, 2018), C1 is not part of an Integrated Health and Social Services Network. The hospital experiences a certain amount of autonomy, it does not have any territorial responsibility towards other facilities, as compared to other hospitals in Montreal. The hospital has an annual budget of more than $410 M.

The second case (C2) is a large hospital in the Montreal Metropolitan Area. This hospital is part of an Integrated Health and Social Services Network (in French, CISSS; University Integrated Health and Social Services Network are called CIUSSS), with a very large catchment in terms of population and geography. This general hospital was created in 1978, and provides, with the Jewish Rehabilitation Hospital, health services to approximately 450,000 inhabitants. As of 2021, C2 had 621 hospital beds and 751 nursing home beds. As of 2015, C2 was composed of 10,357 employees, including 24% of nurses. Its annual budget is about $970 M.

### Ethical considerations

Ethical approval was granted by the Science and Health Research Ethics Board at the University of Montréal for the entire project (CERSES-20-061-D). All interviewees consented to participate in the study and gave their written informed consent.

### Data collection

The first and last authors carried out in-depth interviews via Zoom or by phone, in French, with hospital staff members in both C1 and C2. They applied a purposeful sampling strategy. We aimed to reflect a diverse scope of representations and perceptions of the reassignment decision-making processes, as well as diverse experiences of reassignment (e.g., where reassignment was offered on a voluntary basis, vs. when it was mandatory) and therefore sought to recruit different specialization categories of hospital staff (physicians, nurses, infection prevention control advisors, managers, orderlies, etc.). Interview participants were recruited through contact persons in each hospital, and then through a snowballing strategy. A total of 27 hospital employees participated in a single, one-hour interview (i.e., 15 in C1, and 12 in C2, including about one-third high-level or mid-level managers and two-thirds front-line workers). At the time of the interview, the length of time since reassignment varied across participants. While some of them participated in the research only three weeks after their first reassignment shift, others shared their reassignment experience a few months after. More details about participants and their level of responsibility are available in an ancillary publication (Gautier et al., 2023). Even with a rather small sample for each site, the diversity of interviewed staff in each site (i.e., in terms of position in the hospital hierarchy, and in terms of specialization category as explained above) allowed us to reach empirical saturation, as explained in Hennink & Kaiser (2022). Indeed, after speaking to more than a dozen of key informants in each site (i.e., 27 interviewed staff in total), no additional or new information emerged from the interviews. The conceptual framework for the larger, multicountry research project (Ridde et al., 2021) on health systems resilience guided the development of the interview guide (available upon request).

### Analytical approach

Interviews were recorded, transcribed, and coded using QDAMiner by the first, second, and last authors. Coding was guided by the approach and principles of framework analysis, i.e., using a thematic framework and gradually developing descriptive and explanatory accounts to make sense of the data (Smith & Firth, 2011). In addition, in accordance with the HoSPiCOVID research approach, instead of analyzing each hospital separately, we adopted a comparative perspective. We ensured inter-coding coherence between the three analysts by meeting regularly; these meetings allowed us to develop a common coding tree (available upon request) and to discuss and resolve any analytical issues. These strategies helped to improve the reliability of our coding and the ensuing trustworthiness of our analysis (O’Connor & Joffe, 2020). Three themes were inductively unraveled through this process: 1) Hospitals’ experience of staff reassignment decisions during the first pandemic wave, 2) Individual respondents’ experience with or perceptions of interventions to support reassigned staff's working conditions and wellbeing, and 3) Respondents’ own representations of leadership styles revealed through the reassignment process. We used these emerging themes instead of pre-set analytical categories because we wanted to primarily reflect the *emic* perspective, i.e., the participants’ perspective in their own words. In addition, this analytical approach avoided the risk of producing a dichotomous analysis of complex leadership styles. We translated from French to English all relevant quotes from the original transcriptions; these translations were then revised by a professional English-French translator, and we validated their contents to make sure that they reflected all linguistic nuances.

## Results

### Hospitals’ experience of staff reassignment decisions during the first and second pandemic waves

First, from the beginning of the first wave, the COVID-19 pandemic increased hospital workloads. For several participants in both C1 and C2, the first wave of the pandemic for HHR indeed meant more working hours, which could translate into both physical and “cognitive” fatigue.

Yet COVID-19 did not impact both hospitals in the same way, in terms of number of patients. At the time of massive COVID-19 clusters emerging in long-term care facilities of the Montreal region, C1, being a pediatric hospital, had very few patients, while C2, being a ‘classic’ CISSS hospital covering a large population, was managing surges in COVID-19 patients in its wards. As a result, C1 and C2 experienced the unprecedented provincial decision of staff reassignment to other services and facilities (i.e., the aforementioned communiqué) very differently.

[C1] Since the pediatric clientele is not very affected compared to the adult clientele, it's certain that [in C1], at the beginning, we were very well prepared, […] but we were not as affected, for example, as the long-term care facilities. So, there was a surge of solidarity, […] to help the [wider] health network. […] We started sending our teams to the LTCFs. (KI27, Male, Supply and Logistics department)

In C2, the development of reassignment plans meant that the hospital shifted to a “crisis management” mode:

[C2] When we decided to apply the ministerial orders, and to assign everyone on full time [shifts], and to massively send staff to our public LTCFs, and also to the private LTCFs in the territory. So there, the hospital's staff reassignment plans were implemented, and there we entered […] into a sort of crisis management mode. (KI18, Female, Executive)

The key difference was in the approach taken to reassigning staff, i.e., mandatory or voluntary. At the beginning and prior to the enforcement of ministerial orders, C2 began moving its staff based on a voluntary approach, to facilities overseen by the CISSS with the most acute needs. This approach included the distribution of $1,000 monthly bonuses for staff sent to the long-term care facilities overseen by the CISSS. This volunteering shifted to an obligation when the ministerial orders were enforced. The new obligation entailed the need to mobilize HHR's strongest adaptation skills:

[C2] Not every department can be completely reshuffled after 30 seconds. I'm not sure that everyone had the maximum capacity to adapt. (KI14, Female, Nurse reassigned to Intensive care unit)

By contrast, C1 given its non-integrated status (i.e., not part of a CI(U)SSS), moved its personnel to dedicated hospital services (e.g., its own testing center) and to external facilities with acute needs across different geographical areas of Montreal. High-level managers of C1 would issue calls for volunteers on a pilot basis, which then led to a gradual scaling up to different facilities across Montreal.

[C1] Since we are not part of a CIUSSS, they cannot decide to mobilize us like that, from one hospital to another. […] It was done all on a voluntary basis. They wouldn’t force anyone to-to go there. (KI13, male, Nurse reassigned to long-term care facilities)

[C1] We started with the Geriatric Institute and then quickly they asked us to deploy nursing care resources, so infection prevention advisors and all that, for the entire CIUSSS Central-South Montreal […]. Then from there, we went to help in the East [of Montreal], a LTCF in the East, and then from there in the East, we sent project management teams with the nursing care department, to set up their hot and warm zones and all that, in their LTCFs. (KI15, Female, Nursing care)

The reassignment plan was led by C1's higher-level management teams, in conjunction with the hospital's CEO and heads of departments and units of CIUSSS and hospitals whom they had prior relationships with. In total, 150 C1 volunteers were reassigned to different areas of Montreal over a seven-week period, from April to June 2020. In C2, about 700 staff members were reassigned, the vast majority (about 400) being sent to long-term care facilities overseen by C2 management.

For both C1 and C2 respondents, the staff reassignment created by the COVID-19 crisis also contributed to generating and consolidating a sense of solidarity across facilities in various areas of Montreal.

[C1] You know, [C1 staff] was not only reassigned at CIUSSS Eastern Montreal, […]there were some at CIUSSS Northern Montreal, others at Central-South. The meetings were inter-organizational, our documents were shared, and there was no longer this notion of… of competition, you know, it wasn't, uh… "who manages the COVID better”. […] So I think that COVID has highlighted that [aspect] a lot, the value of collaboration in the health care network. (KI23, Female, Project management)

Such feeling reportedly contributed to motivation and a sense of cooperation for sharing resources and documentation, thus moving beyond competition between facilities. In C2, some respondents noted the support brought by knowledge exchange on infection prevention in the Greater Montreal Region.

### Individual respondents’ experience with or perceptions of interventions to support reassigned staff's working conditions and wellbeing

Because of Bill 10, many of HHRs deployed in long-term care settings were already vulnerable, including professional exhaustion. Both hospitals provided different forms of support that were classified into four categories: i) institutional support tools for preparing and monitoring the reassignment process, ii) post-reassignment processes, iii) peer support tools, and iv) recognition.

#### Institutional support tools prior to and during reassignment

An instrumental element of the reassignment plan for C1 for those sent to long-term care facilities was to offer training to the volunteers prior to their reassignment in LTCFs and private-run long-term facilities. The training lasted two days and included basics of geriatrics and information about how to manage patients’ and their families’ distress and anxiety:

[C1] We decided to train our entire staff ourselves, it's a bit strange that [C1] trained our nurses and some of the network nurses to help them in geriatrics because we’re from pediatrics. But we have set up a whole geriatric training program, for grief, end of life, all that. So, I think that we were leaders in this area. […] There were a lot of people in distress, so we had to deal with a lot of distress too, you know. Preparing [reassigned staff] to manage distress, we… we tried. It's really hard to do that. We tried to prepare them for distress… using videos. (KI15, Female, Nursing care)

In addition to courses on geriatrics and managing grief, the training program included a session on managing staff's own anxiety and psychological distress:

[C1] One of the training sessions was on preventing psychological distress. So, I had created a training session for […] people who were going to a LTCF, […] to prepare them for what they were going to experience, […] and what they could implement during the time of their reassignment to try and keep themselves psychologically healthy. […] We're really in two worlds [pediatric hospital vs. long-term care setting]… So, I was thinking, “I’ve got to find other things”. […] And then I came to look into […] disaster contexts: this was much closer to what we were experiencing. So training designed […] for people who were deployed […] in refugee camps, or in situations where they […] didn't know the environment. […] So, we drew inspiration from humanitarian aid […] because there were… not many things like that, in Quebec. (KI24, Female, Staff health service)

In other words, the respondent sought to collect information from other sources outside of the hospital sector to design their training to manage psychological distress. In particular, they were inspired by the humanitarian sector.

The head of the staff health service thus directly contributed to the training program offered to C1's staff being reassigned to long-term care facilities. The service played a crucial role in supporting reassigned personnel throughout the whole crisis. Together with the coordinator of staff reassignment, the service also implemented systematic and daily follow-up phone calls to every reassigned staff. Individual managers would call their employees to ensure that the reassignment was going smoothly, and that the employee was well supported. In addition, to communicate the basics of infection prevention and control in long-term care facilities, C1's infection prevention team created the job of “COVID agent”. Those COVID agents were sent along with reassigned staff as additional support, to set up red, orange, and green zones in LTCFs.

C2 implemented “basic training” for staff sent to intensive care units and/or long-term care facilities, as well as a special hotline for reassigned staff who needed advice and/or psychological counseling. However, institutional support structures were not as proactive. The training for reassigned personnel was considered too hasty, and the hospital did not have a staff health service as in C1. In addition, because C2 hospital managers were themselves reassigned, monitoring and follow-up communication with their employees was unfeasible. Therefore, once reassignment started, links could not be maintained between reassigned staff and their managers, who were themselves often reassigned to other facilities or units. In general, the lack of managerial continuity, including communication during the reassignment, raised criticism. Reassigned staff notably deplored the fact that the person managing communications on reassignment (e.g., who, where) was continuously changing. After a few weeks, C2 leadership decided to appoint a new head of department, responsible for ‘the reassigned’, as well as a team of psychosocial workers dedicated to reassigned staff:

[C2] We put in a head of the department, [a department] which we called “the reassigned”, so already, there was a resource person to refer to, uh… when there was a problem. […] So, we appointed a manager for the reassigned, […] then, well, we set up a team of psychosocial workers, both in the LTCFs but even afterward, for the staff who needed support. (KI18, Female, Executive)

These interventions did not appear to fully meet the needs (i.e., adequate training and preparedness prior to reassignment), possibly because they were implemented too late in the process. After the first COVID-19 wave, the management of reassigned staff was discussed at length by C2's top leadership, with the desire to improve support to reassigned staff, particularly psychological support.

Respondents from C1 reported some other measures implemented by the hospital leadership to protect reassigned staff against COVID-19 infection:

[C1] I went to the Geriatric Institute to look into infection control with my infection control nurse. […] We made sure that where they went, there was enough personal protective equipment for them, and for the rest of the team. We provided faceshields for everybody, […] masks. [Our] clear recommendation [was] that if they were missing personal protective equipment, our staff would leave. And since the management didn't want them to leave, everybody made sure that there was enough [PPE] for everybody […]. (KI01, Female, Infection prevention and control unit)

Despite these preparations and training, the lack of PPE in LTCFs where C1 and C2 staff were sent to, had consequences on their physical health, COVID-19 infection, and their mental health, notably strong fears of becoming infected and feelings of exhaustion.

#### Post-reassignment support processes

For those reassigned, returning to their original posting in hospitals was difficult. In C1, they would realize how different their experience was, in comparison to that of their non-reassigned colleagues:

[C1] The reassigned staff, most of them, they were [in LTCFs] for six weeks. And they’d come back six weeks later, and their environment is completely disconnected from what they’ve experienced. Because here [at C1] we had… protected areas, never fearing of running out of personal equipment… […] The shock of returning…, […] that was the hardest part. (KI24, Female, Staff health service)

In addition, many reassigned staff experienced psychological distress with some having experienced trauma due to the dire circumstances of LTCFs (e.g., fear of COVID infection, mourning the death of many long-term care residents). For the staff health service at C1, it was crucial to anticipate this issue: they created another short video (45 min) to prepare the reassigned staff for when they’d get back, and organized group sessions, as well as debriefing meetings, where they would talk about the “shock of the return” based on the videos. In addition, a complete, “careful debriefing” was organized upon return to facilitate the staff's reintegration into the hospital. Such debriefing involved the training of managers and people in charge of supervising/organizing the post-reassignment process, and the design of a post-reassignment and reintegration support system.

[C1] In preparation for the return [of reassigned staff], we created a ten-minute video for […] managers who had reassigned employees, on how to support those employees upon return. To ensure that [the managers] know what the symptoms of post-traumatic stress disorder are, to ensure that they know how to identify them, like: “offer some time, you know, you can't just expect [your employee] to be able to tell you about their experience in three minutes. Take some time in your calendar, propose a meeting, then ask them, you know, to tell you”… So, we created that [video] to ensure that managers were equipped to receive people […] in their team. (KI24, Female, Staff health service)

C1 interviewees noted that the reassigned staff had the feeling of being well-supported during the post-reassignment process.

In C2, staff experienced worse conditions as they returned to their original hospital units because the reintegration process was not anticipated and prepared as much as it was in C1. Most importantly, in C2, apart from granting a few days of holidays (when it was possible) and offering access to a team of psychosocial workers, addressing the aftermath of the psychological shock associated with reassignment was not considered a priority in the recovery and reintegration plans:

[C2] So […] we managed the return [of reassigned staff]. I coordinated the… sequence of recovery plans for the departments I’m responsible for, […] and we said to the committee, “here are the stages, now [you need to] arrange the return of staff according to these plans”. They met each day to plan who would go where, and in what order […]. Every week, up to today… […] the directors are asked to update the next phases of recovery, to know if the resources are still deployed [somewhere] or if they need to return on-site. (KI18, Female, Executive)

A C2 manager stated that the negative impacts of HHR reassignment persisted, noticing numerous resignations from staff who had been reassigned and whose specialty was in high demand, which was particularly problematic. Resignations also came from some of the non-reassigned staff, who also had experienced organizational changes (e.g., their clinical team being completely rearranged with additional or fewer staff). In response to this, interviewees highlighted the importance of organizing frequent follow-up meetings with managers.

#### Peer-support tools

In both C1 and C2, executives implemented a peer work system: individual reassigned staff were assigned to another reassigned staff who would support one another throughout the reassignment process. Some C2 staff expressed mixed feelings toward that configuration:

[C2] They put us in ‘duos’; so they asked us to help, so we weren't really in charge of the patient: we were like a team. But… it all depended on who we had as a partner […]. For people like me who are used to their autonomy, complete [autonomy], […] it required more… […] adaptation […] to let someone else take charge, […] to somehow give up control. (KI14, Female, Nurse reassigned to Intensive care unit)

In C1, the peer work system was called ‘battle buddies’, a concept borrowed from the humanitarian sector:

[C1] It's like the kind of idea of “you're like in a war zone when you're in a pandemic zone”. […] You must “find your battle buddy, the person that you go with”. And then, you know… you keep a link every day, you make sure that the other one is ok. […] So it was about trying to create links with someone who's going to be there for you, […] just having that mutual support. (KI24, Female, Staff health service)

Such a peer work system paved the way for more spontaneous peer bonding and information sharing, e.g., a Facebook page for the reassigned staff.

#### Recognition

Several strategies were developed to highlight the significance of reassigned staff efforts. Individual bonuses were provided to reassigned staff. C2 also sent letters of recognition to all reassigned staff upon their return. On social media, a ‘*campagne de reconnaissanc*e’ (recognition campaign) for HHR (particularly those who had been reassigned) was also implemented. In C1, the head of the staff health service also spontaneously created more personalized forms of interaction with reassigned staff:

[C1] You know [the head of the Staff health service] she was exceptional, she’d call [reassigned staff], she’d send them flowers… […] She went out of her way to like, show that we cared. […] She really managed the human side of like… […] She's truly exceptional. She was the kind of person who, when there was a [COVID-19] case, she’d call, she’d follow up, she’d take care of people. (KI22, Female, Nursing care)

Other respondents highlighted how C1 staff were privileged to have such support. Such strategies were perceived as strengthening the staff's sense of security and confidence within the organization.

### Respondents’ own representations of leadership styles revealed through the reassignment process

Interviewees often offered their own insights and analyses of the reassignment decision-making and ensuing support process, referring to different leadership styles. From an organizational structure perspective, Bill 10 was perceived as introducing a more top-down, highly-centralized, decision-making model. According to interviewees, the reform introduced a top-down structure that also acted as a major predisposing factor in terms of leadership. More specifically, for our participants, whether the hospital was integrated into CI(U)SSS or not, allowed for the expression of certain features of leadership styles. Those features could be broken down roughly into two categories: top-down ‘authoritarian’ and ‘*laissez-faire’* forms of leadership on the one hand, allowing for inequalities to emerge, and, on the other hand, “maternal leadership style”.

First, the implementation of Bill 10 incurred a reinforced centralized decision-making model at the level of CI(U)SSS. The fact that reassignment took place in such an organizational context, led to the expression of a top-down HHR management style (e.g., in the communication approach). This was often illustrated in C2 interactions between the CISSS executives and front-line workers. Executives indeed had to enforce Ministry's orders (e.g., on staff reassignment to long-term care facilities):

[C2] [W]e received orders, we knew what had to be put in place, we were in a top-down configuration, so we executed [the orders]. […] Because we often heard that ‘the CEOs don’t listen to the Ministry's orders’, et cetera. I’d say that [at C2] the orders were followed to the letter. (KI18, Female, Executive)

This phenomenon was considered as translating a top-down, authoritarian form of leadership, which mirrored the Ministry's centralized decision-making. It received much criticism from C2 staff. Relations between high-level managers and unions reportedly worsened in the context of a highly centralized decision-making. Unions notably criticized the fact that decisions were made outside the purview of collective agreements:

[C2] Well, with the ministerial order, we heard of it before it arrived. We read it, and the employer [at C2] met with our union. Then [Executives at C2] told us: ‘We’ll consult you and then we’ll avoid using the ministerial order, and then we would warn you in advance if we needed to use it’. In the end, they decided on a certain day without telling us that it was going to happen. […] So, [this ministerial order] sets the tone […] of like, dictatorship. From then on, it was decided that the collective agreements would no longer be used. […] We can send you where we want, where we need. (KI12, Female, Lab unit)

This top-down approach coincided, on the ground, with a form of *laissez-faire* leadership. Reassigned staff indeed highlighted a feeling of ‘abandonment’ in LTCFs, going hand-in-hand with local facilities managers’ absence:

[C2] They decided to take them all [the employees] and send them to the LTCFs, but the LTCFs were not even ready to receive them. […] It was really chaos from the beginning. It was like there was no real leader who had said: ‘you, managers in every location [of reassignment], you’ve got to be there, you’re going to do it this way’. (KI12, Female, Lab unit)

According to some C2 interviewees, front-line workers “were already fragile” because of the 2015 reform, which had induced a lack of organizational support (i.e., many mid-level management positions being cut).

Reassigned staff from C1 also described leadership peculiarities specific to the nature of the pediatric hospital. For instance, the sharing of equipment and resources was considered a feature of the servant form of leadership that seemed to dominate in C1. Some female and male respondents even spoke of a form of “maternal” leadership at C1, implying that the hospital's managers, a majority of them being women, made sure to stay close to their team members/unit staff all along, regularly checking in and listening to their needs and concerns. This feature appeared to go beyond the pandemic or crisis context, it was put in parallel with the origins and core mission of the hospital, traditionally run by women:

[C1] [C1] you know, in its DNA… […] [C1] has always been a hospital most often managed by women. […]. It seems to be part of the organization's humanity. […] That's part of the reason why I stayed here, honestly. Because… […] You know, the-the maternal side – well, that's reductive – […] the-the caring side… which women have more. […] The fiber at [C1] is a maternal fiber. (KI05, Male, Pharmacy department)

According to several respondents, the “maternal” style led to creating a positive supportive environment, which developed a certain sense of pride and renewed belonging among staff – a feature that could, in turn, mirror the inclusive leadership style. Some interviewees reckoned that in CI(U)SSS, mostly run by men, the support structures might be less ‘attentive’:

[C1] I also find it super inspiring to work with women like LP, like KT, who are super high [in the hierarchy] but who are super human, super close to their employees […]. You know [C1], it's a lot of women, but in the CIUSSS there were a lot of men, and… I think it's a beautiful side of women leaders to be perhaps more human, and more attentive to others, than men can be. And I know, I'm generalizing big time here, but… (KI22, Female, Nursing care)

Some middle- and high-level managers yet noted some of the limitations of this “maternal” leadership model, particularly in relation to the personalized support offered to certain staff. Some interviewees stated that it could restrain the development of autonomy among certain members of the hospital's staff. In response, managers are calling for an evolution of an organizational culture that would enhance staff autonomy, while maintaining a human approach to management of HHR. This form of leadership, which high-level and mid-level managers aspire to, appeared very close to that of ‘caring leadership’. Based on interview findings, Table 2 offers a summary of the key themes and sub-themes for each case.

## Discussion

Our study is amongst the few empirical research investigating relationships between leadership styles of health care organizations and organizations’ preparedness or response adequacy towards HHR in times of crisis. Based on our case study on two hospitals (C1 and C2), we documented features of diverse leadership styles revealed through the experience of support tools made available to HHR reassigned to long-term care settings in the context of the first and second waves of COVID-19.

**Table 2.** Summary of key themes and sub-themes for each case.

	Case 1 (non-integrated)	Case 2 (CISSS-integrated)
**Organization’s governance** **structure**	Autonomous	Reporting to ministry; ministry-appointed board of directors
**Reassignment decision**	Voluntary	Voluntary, then compulsory
**Tools for staff reassigned preparedness**	Training in geriatrics and for managing grief and PTSD	training
**Tools to support reassigned staff**	Daily 1-1 check-in phone calls; training and sending of ‘COVID agents’; dedicated hotline; dedicated service with stable support staff; ‘battle buddies’; Facebook page for peer support	Team of psychosocial workers dedicated to reassigned staff; dedicated hotline; dedicated department but lack of management continuity; peer work
**Tools for post-reassignment support**	Debriefing; post-reassignment session upon reintegration in the hospital; training sessions for the managers of reassigned staff	Team of psychosocial workers dedicated to reassigned staff
**Incentives and rewards**	Bonuses; sending flowers	Bonuses; letters of recognition; social media recognition campaign
**Perceived leadership style**	‘Maternal leadership’ (e.g., health staff; approachability of female high-level managers)	Authoritarian leadership (communicating reassignment decisions with no discussion) *Laissez-faire* leadership (i.e., leadership virtually absent on the ground)
**Leadership aspirations for the future**	Moving towards caring leadership to enhance staff autonomy	Moving towards transformational, inclusive leadership

Source: Authors’ own work

At the start of the pandemic, both hospitals were facing pre-existing chronic HHR shortages. These shortages were reinforced by the pandemic crisis, because of the isolation and quarantine policies for any employee returning from travel and/or having contracted COVID-19 or being in contact with cases. Yet, C1 and C2 handled their HHR reassignment very differently. In one case (C1), the relatively low COVID-19 patient flows and a largely autonomous organizational structure, allowed for voluntary reassignment, which was not possible in CI(U)SSS-integrated hospitals like C2. As shown in Table 2, in C1, support systems included training in geriatrics and psychological distress management, regular telephone follow-ups with reassigned staff, battle buddies, and a debriefing and post-reassignment session upon reintegration in the hospital. In C2, reassigned nurses and other hospital staff had access to basic training, could be paired with someone of their occupation (pair working), and could call for assistance from a special psychosocial reinforcement team, or asked for advice via the use of a dedicated hotline. The design and handling of the reassignment system, as well as the different strategies to support reassigned staff, revealed C1 leadership's strong will to intervene and control the process in a compassionate or ‘caring’ fashion, which interviewees characterized as reflecting a certain “maternal leadership”, appeared to be conducive of a successful crisis response. There are important considerations, in addition to low caseloads (which notably allowed for more preparedness time), such as autonomous governance which enabled less dependence on Ministerial orders, more fluid decision-making, and the liberty to choose how to use their PPE stocks and how to allocate their HHR.

In C1, several characteristics of leadership described as “maternal” by interviewee respondents, allowed us to connect it to transformational leadership – particularly the “caring” feature. Similarly, Panton describes maternal leadership as “transformational leadership… with a pedagogy of caring” (Panton, 2016, p. 23). In our study, the maternal leadership style also shared commonalities with several subtypes of transformational leadership, such as the inclusive leadership style, in that it allowed for strengthening trust and the collective sense of belonging for HHR reassigned on the frontlines (Jambhekar, 2019). In addition, the “maternal” type seemed very close to compassionate managerial leadership, i.e., a leadership style that promotes organizational strategic goals (survival, sustainability) “while also addressing employees’ concerns (wellbeing, fear, uncertainty), through leadership compassion, benevolence, empathy, caring, engagement, reassurance and motivation” (Oruh et al., 2021, p. 1364). As in the case of the compassionate managerial leadership style, the support systems implemented by C1 leaders helped to alleviate stress experiences among HHR during and after reassignment, which in turn seemed to the hospital's resilience (Ridde et al., 2021).

Elements of servant leadership were also salient in the case of C1. Key attributes of servant leaders (Allen et al., 2016) that would bring them close to the “maternal” style, included leaders’ listening abilities (highlighted by several respondents), their inclination to promote teamwork and collective problem-solving, as well as their capacity to motivate by providing autonomy (e.g., promote a sense of initiative) and resources (e.g., PPE in LTCFs). Timely provision of adequate material resources (e.g., PPE) and implementation of mental health supports are in fact likely to increase staff self-efficacy and confidence (Wu et al., 2020). Regulatory bodies also recommended that hospital managers be trained to prevent and detect the onset of distressing symptoms in their staff, particularly for those reassigned to other care settings (INESSS, 2020). Again, the described experience of C1, which developed a training program for managers whose staff had been sent to LTCFs during the spring 2020 crisis, and for the staff themselves, was particularly illustrative. Some of the videos on how to cope with psychological distress and prevent post-traumatic stress disorder that they developed were transferred to other hospitals in Quebec. Beyond the individual resilience of health care personnel, these strategies could therefore strengthen organizational resilience (Behrens et al., 2022).

By contrast, leadership in C2 displayed more centralized and vertical decision-making, and much less proactive intervention, often coming up with support tools considered too basic, too little, or too late – especially in the context of HHR already being vulnerable. This reflects the impact of the 2015 reform; the pandemic creating multiple situations whereby leadership teams were caught between two sides (i.e., the ministry's orders and HHR wellbeing). Specifically, insufficient or late reactions were noted in C2, such as poor communication on the implementation of reassignment (managers vs. professionals, CISSS vs. unions), and the lack of consideration for employees’ mental health in general, or the lack of preparation for the “re-integration” process. The choice of passive interventions to support reassigned staff, mirroring a form of transactional leadership, may have been costly for C2. C2's reassigned staff strongly expressed the need for additional moral and psychological support from the institution. This is coherent with one of the key recommendations provided in a major report on health systems resilience across the globe, “health workers need support too” (Sagan et al., 2021, p. 93). In C2, staff specifically asked for more proactive support (i.e., offering support rather than giving it on demand). As a result, the difficult management of reassigned staff hindered the quality of the response to the pandemic crisis. It also had negative mid-term impacts on the hospital. Indeed, the transfer of professionals to completely different environments may have had significant impacts at the beginning, and then persisted through time. This led to several resignations, particularly in professions in short supply.

Our study also revealed a nuanced portrait of leadership styles. Indeed, while the key themes highlighted contrasted leadership styles between C1 and C2, they also showed diversified forms of leadership through the reassignment experience. For instance, both C1 and C2 included forms of transactional leadership (i.e., incentive and reward system – see Table 2). In C2, authoritarian leadership appeared to co-exist with forms of *laissez-faire* leadership on the ground. In C1, “maternal leadership” coincided with managers’ wish to move towards caring leadership in order to further staff autonomy. More broadly, our findings emphasized the complex nature of organizational leadership in the context of unpreceded circumstances (the COVID-19 crisis).

The COVID-19 pandemic further highlighted the importance of human work (Behrens et al., 2022) which is at the core of hospitals and health systems resilience. In April 2020, the *Institut national d'excellence en santé et en services sociaux* of Quebec published a rapid review of the literature that highlighted the main sources of psychological distress resulting from the COVID-19 pandemic for health human resources (INESSS, 2020). In addition to pandemic-related risks, the review noted that factors related to organizational environments may exacerbate psychological distress or mental health status among HHR. In fact, one of the key factors in the prevention of mental health problems in the context of COVID-19 included strong leadership within health care facilities (Wu et al., 2020). The current HHR situation in Quebec has significantly worsened since 2020, the pandemic accelerating an already difficult situation. Even though the pandemic is not over, we can suggest that, at least on the HHR front, Quebec's health system demonstrated a rather low resilience, since the situation only worsened for HHR since the beginning of 2020. Closed emergency rooms and postponed surgeries have been recurrent since 2021 because of workforce shortages, leaving institutions no choice but to implement “contingency plans” or crisis management solutions, and to hire more personnel through private placement agencies. A vicious circle seems to be in place, with a lack of resources leading to worsening working conditions and resulting in poorer retention (The Canadian Press, 2022). To address these challenges, Quebec's government announced a plan to rebuild its healthcare system, with HHR being one of its top priorities ( ICI.Radio-Canada.ca, n.d.). The ambition is to move “from understaffing to overstaffing”, e.g., by bringing in 1,000 nurses from abroad and recruiting 3,000 administrative officers (*ibid.*). In terms of staff retention, this plan provides for the implementation of self-scheduling, based on the success of several pilot projects in some places. Those measures may sound promising, but with the crucial question of managerial leadership styles remaining a blind spot for policy-makers, the ambitious plan might not yield the expected effects, particularly in terms of staff retention.

This study also offers insight into several recommendations to support the mental health and resilience of the health workforce. At the macro-political level, more consideration for adaptive and agile decision-making in hospitals in times of crisis should be given when designing new health system policies, a recommendation that is aligned with the global report on health systems resilience (Sagan et al., 2021). This could be conducive to the expression of a more diverse set of leadership styles with the goal of bringing hospital leaders closer to hospital staff's needs (e.g., more person-centered leadership styles, such as the servant leadership style). At the organizational level, systematizing the use of anticipatory planning tools (e.g., needs assessment) in hospitals would entail better organizational preparedness when facing a crisis (Dagenais et al., 2023; Gabet et al., 2023). Beyond times of crisis, given the importance of maintaining close links with teams on the front line, implementing a staff health service – another element that we can link to servant leadership – would certainly provide a sustainable and proactive response to HHR's support needs. If such service cannot be considered an option, e.g., for financial reasons, specific provisions for HHR supportive communication systems in hospitals should be anticipated in yearly budgets. More broadly, the generalization of graduate or practice-based teachings about leadership styles could be an interesting option to consider for hospital managers, at least to expose them to the diversity of leadership styles, to their value and limitations. Lastly, involving mid-level managers of health care facilities more actively in the collective identification and formulation of a ‘toolbox’ of solutions to support mental health at work, would be most valuable (Corbière et al., 2020).

This study has several limitations. Data collection was carried out in Summer and Fall of 2020 (i.e., after the first wave of COVID-19, and during the second wave), reflecting different pandemic timeframes, and changing perceptions for interview respondents, who were HHR themselves going through those changes. The fluctuating pandemic context also reflected a discontinuous and sometimes incoherent decision-making process in times of crisis, which was often difficult to reconstruct post hoc. For these reasons, our study findings ought to be interpreted in their respective time and space, which might challenge the possibilities of extending the findings to other settings.

## Conclusions

As with previous studies, our study has demonstrated that many aspects of Quebec's pandemic response are related to the major political reform of 2015. By re-enforcing a top-down, highly centralized decision-making, this health systems reform shaped hospitals’ manifestation of certain leadership styles. C1, not being under direct control by the ministry (i.e., a non-integrated hospital) was not bound by the ministerial reassignment order. Executives in C1 were thus free to proactively design support systems and offer reassigned HHR timely and adequate psychosocial support tools. For leaders of C2, which belonged to a CISSS, lack of compliance with ministerial orders was not an option, even if that resulted in creating long-standing tensions within the hospital's HHR.

Our study also offered several insightful recommendations to support the mental health and resilience of the health workforce in times of crisis and beyond. As the retention of HHR becomes an even more priority issue for policy-makers, and since future crises will certainly unfold in the next few years, we also encourage the production and diffusion of more research in other Canadian provinces on wellbeing support tools at work for nurses and health care professionals.
